# Fine needle aspiration of pancreatic lesions focusing on secondary tumors with emphasis of metastatic breast cancer: A clinicopathological study with follow‐up

**DOI:** 10.1002/cam4.5374

**Published:** 2022-10-24

**Authors:** Maria Yanqing Chen, Neda Zarrin‐Khameh, Ya Xu

**Affiliations:** ^1^ Department of Pathology & Immunology Baylor College of Medicine Texas Houston USA; ^2^ Department of Pathology & Immunology, Baylor College of Medicine, Department of Pathology, Ben Taub Hospital, Harris Health System Texas Houston USA

**Keywords:** biomarker, fine needle aspiration, metastatic breast cancer, pancreas, secondary tumor

## Abstract

**Background:**

The data on metastatic tumors to the pancreas diagnosed by fine needle aspiration (FNA) biopsy is limited. We report our experience of FNA of primary and secondary pancreatic tumors emphasizing metastatic breast cancer in the pancreas.

**Method:**

Total 274 cases of pancreatic FNA in 10 years were retrospectively reviewed. Literature review of metastatic breast cancers to the pancreas was performed.

**Results:**

Out of the 274 cases, 7 (7/274, 2.6%) cases were non‐diagnostic, 46 (46/274, 16.8%) cases were negative for malignancy, and 40 (40/274, 14.6%) cases were under the category of atypical cells. There were 133 (133/274, 48.5%) cases diagnosed as positive for malignancy, 20 (20/274, 7.3%) suspicious for malignancy, and 28 (28/274, 10.2%) cases in the category of neoplastic: other. The most common neoplasm diagnosed was ductal adenocarcinoma (114/274, 41.6%; 114/133, 85.7% in positive for malignancy category). Ten cases (10/274, 3.7%) were diagnosed as metastatic neoplasms to the pancreas, including four breast infiltrating ductal carcinomas (IDC), one endocervical adenocarcinoma, one anal/rectal squamous cell carcinoma, one renal cell carcinoma, one hepatocellular carcinoma, one seminoma and one lung adenocarcinoma. We summarized the biomarkers of the four metastatic breast cancers and conducted literature review on biomarkers of metastatic breast cancers to the pancreas.

**Conclusions:**

Upon analyzing FNAs of primary and secondary tumors in the pancreas, we have found breast carcinoma is the most common secondary pancreatic neoplasm in our patient population. Triple negative breast ductal carcinoma is the most common tumor among the metastasis of breast carcinomas to the pancreas. To the best of our knowledge, this study is the first report with a literature review focusing on biomarkers of metastatic breast cancer to the pancreas.

## Graphical abstract

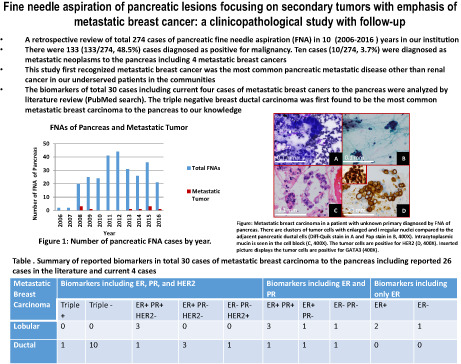

## 1 INTRODUCTION

Imaging guided fine‐needle aspiration (FNA) is an indispensable tool in diagnosis of pancreatic masses. Over the past several decades, endoscopic ultrasound guided FNA (EUS‐FNA) has been shown to be safe and effective, and has mostly replaced other imaging‐based methods such as magnetic response imaging or computed tomography guided FNAs as the preferred method for cytologic evaluation of pancreatic lesions.^1^, ^2^, ^3^ The Papanicolaou Society of Cytopathology has developed a set of guidelines for pancreatobiliary cytology, published 2014,^4^ and these guidelines are used in this study retrospectively.

More than 85% of solid pancreatic tumors are ductal adenocarcinoma.^5^ Pancreas is a rare site for tumor metastasis. Neoplasms that most often metastasize to the pancreas include carcinomas of the kidney, lung, skin (melanoma), breast, gynecologic tract and gastrointestinal tract.^6^ To diagnose primary tumors and metastases in pancreas by FNA is challenging. In this retrospective study, we have examined 274 cases of pancreas lesions diagnosed by FNA from 2006–2016 (pancreatic FNAs largely replaced by small core biopsy after 2016 in our institution), and found 10 cases of metastases to the pancreas. Among these 10 cases, there are four cases of metastatic breast cancer. We also have analyzed the biomarkers in the metastatic breast cancers to the pancreas by literature review.

## 2 METHODS

This is a retrospective study involving a total of 274 cases of pancreatic CT‐ or endoscopic ultrasound (EUS)‐guided fine needle aspiration (FNA) procedures taking place between 2006 and 2016 at a tertiary medical center (FNA of pancreas was largely replaced by EUS‐guided small core biopsy after 2016 in our institution). The cases were identified through a search of our institution's electronic pathology database. On‐site cytology evaluation was performed for each case. Slides were prepared by using Diff‐Quik and Papanicolaou stains. Pertinent immunohistochemical stains were performed on cell blocks. Four patients underwent a second FNA procedure for confirmation and the diagnoses were updated from atypical cells or suspicious for malignancy to mucinous neoplasm, acinar cell carcinoma and two ductal adenocarcinomas, respectively. All FNAs were counted toward the total number of FNA cases. Other clinical information, such as demographics and clinical course, were gathered from the electronic medical record database. All cases were re‐evaluated by using the guidelines for pancreatobiliary cytology published by Papanicolaou Society of Cytopathology in 2014.^4^

The biomarkers of the metastatic breast cancers to the pancreas were further analyzed by literature review (PubMed search for metastatic breast cancers to the pancreas). There were total 51 reported cases including our four cases of metastatic breast carcinomas to the pancreas, with 22 lobular and 29 ductal carcinomas. Among these 51 cases, 30 cases including current 4 cases had reported biomarkers (references were in the discussion section). The information of biomarkers on these 30 cases was summarized in Table 4.

## 3 RESULTS

**FIGURE 1:**
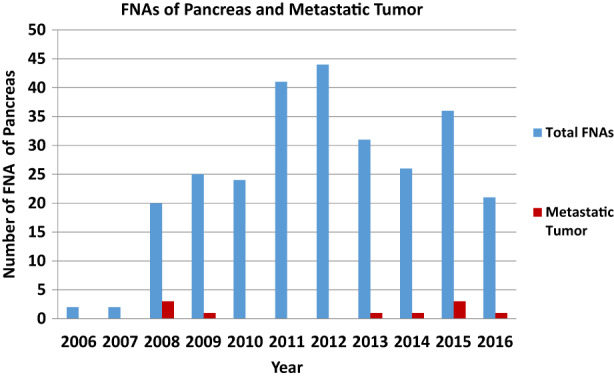
Number of pancreatic FNA cases by year

A total of 274 cases of pancreatic FNAs were studied in a period of 10 years (2006 to 2016). Over this time, the number of pancreatic FNA procedures steadily increased from 2 cases in 2006 to a peak of 44 cases in 2012 (Figure 1).

The average age of patients who received FNA procedures was 56.8 years (range from 20 to 89, standard deviation of 11.9). There were 118 males (47.6%) and 130 females (52.4%). We analyzed all of the cases by using the guidelines for pancreatobiliary cytology published by Papanicolaou Society of Cytopathology in 2014.^4^ As shown in Table 1, out of the 274 total cases, 7 (7/274, 2.6%) cases were non‐diagnostic, 46 (46/274, 16.8%) cases were negative for malignancy, and 40 (40/274, 14.6%) cases were under the category of atypical cells. There were 133 (133/274, 48.5%) cases diagnosed as positive for malignancy, 20 (20/274, 7.3%) suspicious for malignancy, and 28 (28/274, 10.2%) cases in the category of neoplastic: other. And 161 (133 + 28) cases had definitive diagnoses. When evaluated by the individual diagnosis, the most common neoplasm diagnosed was ductal adenocarcinoma (114/274, 41.6% among all FNAs; 114/133, 85.7% in the category of positive for malignancy), followed by neuroendocrine neoplasm (16/274, 5.8%), metastasis to the pancreas (10/274, 3.7%), solid pseudopapillary tumor (6/274, 2.2%), mucinous neoplasm (5/274, 1.8%), poorly differentiated carcinoma (2/274, 0.7%), adenosquamous carcinoma (2/274, 0.7%), undifferentiated carcinoma (2/274, 0.7%), acinar cell carcinoma (1/274, 0.4%), intraductal papillary mucinous neoplasm (1/274, 0.4%), non‐Hodgkin lymphoma (1/274, 0.4%), and direct extension of ampullary adenocarcinoma to pancreas (1/274, 0.4%). Ten cases (10/274, 3.7%; 10/133, 7.5% in the category of positive for malignancy) were diagnosed as metastatic neoplasms to the pancreas. Table 1 summarized the findings.

**TABLE 1:** Summary of the cytopathology diagnoses of 274 FNAs of pancreatic lesions on 248 patients (some patients received multiple FNAs and/or had multiple lesions*)

Diagnosis	# of cases (%)		# Male patients	# Female patients
Non‐diagnostic/Unsatisfactory	7 (2.6%)		2	4*
Negative for malignancy	46 (16.8%)		22*	21*
Atypical cells	40 (14.6%)		11*	13*
Neoplastic: Other	28 (10.2%)			
Solid pseudopapillary tumor	6 (2.2%)		0	6
Mucinous neoplasm	5 (1.8%)		0	5
IPMN	1 (0.4%)		1	0
Neuroendocrine neoplasm	16 (5.8%)		8*	6*
Suspicious for malignancy	20 (7.3%)		11	6*
Positive for malignancy	133 (48.5%)			
Ductal adenocarcinoma	114/274 (41.6%)	114/133 (85.7%)	53	60*
Acinar cell carcinoma	1 (0.4%)		1	0
Poorly differentiated carcinoma	2 (0.7%)		1	1
Adenosquamous carcinoma	2 (0.7%)		1	1
Undifferentiated carcinoma	2 (0.7%)		2	0
Non‐Hodgkin lymphoma	1 (0.4%)		1	0
Ampullary adenocarcinoma with direct extension to pancreas	1 (0.4%)		0	1
Metastasis to pancreas	10 (3.7%)		4	6
TOTAL	274 cases		118	130
			248 patients	

Abbreviation: IMPN, intraductal papillary mucinous neoplasm.

**FIGURE 2:**
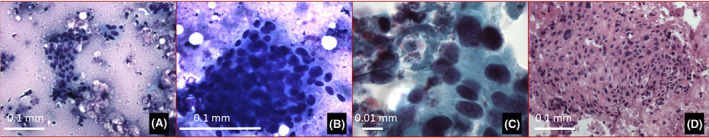
Metastatic squamous cell carcinoma (SCC) in a patient with a history of anal/rectal SCC. Cohesive clusters of tumor cells with dense cytoplasm are present. (Diff‐Quik stain: A, 200X; B, 400X). Intercellular bridging is seen under high power (C, Pap stain, 1000X). The cell block has pleomorphic tumor cells (D, 200X).

**FIGURE 3:**
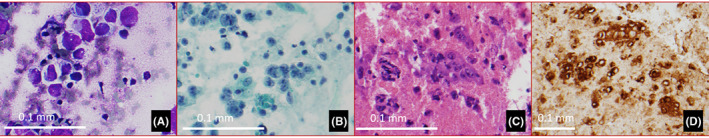
Metastatic seminoma in a patient with a history of testicular seminoma. There are discohesive tumor cells with enlarged nuclei intermixed with lymphoid cells (Diff‐Quik stain in A and Pap stain in B, 400X). Tigroid background is present in A. Prominent nucleoli are seen in B. The cell block has scattered tumor cells (C, 400X). The tumor cells are diffusely positive for PLAP (D, 200X).

Six out of the ten metastases to the pancreas were endocervical adenocarcinoma, hepatocellular carcinoma (HCC), lung adenocarcinoma, anorectal squamous cell carcinoma (SCC) (Figure 2), clear cell renal cell carcinoma (RCC), and seminoma (Figure 3). The ages of these six patients ranged from 35 to 59 years, with two females and four males. The interval of diagnosis of the primary tumor to the diagnosis of metastasis in the pancreas ranged from 4 months to 8.6 years, except one patient with HCC had synchronous diagnoses of primary cancer and pancreatic metastasis. The most common symptom was jaundice. These six patients except the patient with HCC had confirmed metastasis in other organs. After the diagnoses of pancreatic metastases, two patients with lung adenocarcinoma and endocervical adenocarcinoma died 1 month and 2 months later, respectively. Two patients with HCC and anorectal SCC, respectively, were placed in hospice care several months later. The patient with metastatic seminoma was lost to follow‐up after achieving remission. The patient with RCC was still living at the time of this report, with stable disease for 13 years. The above findings are summarized in Table 2.

**TABLE 2:** Cases of metastatic cancer other than breast primary to the pancreas diagnosed by FNA

Primary diagnosis	Age/gender	Location of pancreatic mass & size by imaging	Main symptoms	Other metastatic sites	Interval of pancreatic metastasis and primary tumor	Follow‐up time from Dx of pancreatic metastasis
Endocervical adenocarcinoma	45/F	Neck, 5.7 cm	Nausea, vomiting	Lung	5 months	2 months (died)
Hepatocellular carcinoma	59/M	Head, 6.5 cm	Painless jaundice	None	Synchronous	3 months (hospice, lost to follow up)
Lung adenocarcinoma	47/M	Head, 3.5 cm	Painless jaundice	Brain	4 months	1 month (died)
Anorectal Squamous cell carcinoma	54/M	Head, 3.5 cm	Painless jaundice	Lung	5.3 yrs	2 months (hospice, lost to follow up)
Clear cell renal cell carcinoma	58/F	Tail, 6.0 cm	None	Liver	8.6 yrs	13 years (stable)
Testicular Seminoma	35/M	Head, 3.5 cm	None	Peritoneum	3 yrs	Remission ‐ 1.6 yrs (lost to follow up)

Abbreviations: Dx, diagnosis; yrs, years.

**FIGURE 4:**
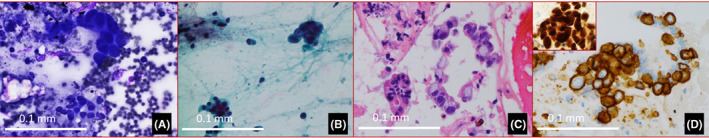
Metastatic breast carcinoma in a patient (Patient IV) with unknown primary. There are clusters of tumor cells with enlarged and irregular nuclei compared to the adjacent pancreatic ductal ells (Diff‐Quik stain in A and Pap stain in B, 400X). Intracytoplasmic mucin is seen in the cell block (C, 400X). The tumor cells are positive for HER2 3+ (D, 400X). Inserted picture displays the tumor cells are positive for GATA3 (400X).

The other four of ten cases (40%) of the metastases to the pancreas were of breast origin (Table 3). The ages of the four female patients ranged from 37 to 64 years old. Three patients (Patients #I‐III) had breast infiltrating ductal carcinoma (IDC) for 1 to 21 years prior to the diagnosis of pancreatic metastasis. The fourth patient (Patient #IV) without cancer history was found to have breast mass at presentation (the diagnosis of IDC was later confirmed). The biomarkers included estrogen receptor (ER), progesterone receptor (PR), and human epidermal growth factor receptor2 (HER2) were analyzed. Triple negative IDCs were found in two patients (Patients #II & III). The IDCs in other two patients (Patients I & IV) were ER+, PR‐, HER2 3+ and ER‐, PR‐, HER2 3+, respectively. For treatment, patient #I received lumpectomy, chemotherapy (Chemo), radiotherapy (XRT), and patient #II underwent neoadjuvant chemotherapy, total mastectomy, and Whipple surgery/pancreaticoduodenectomy for pancreatic metastasis. Patient #III had neoadjuvant chemotherapy, intended lumpectomy (not performed), Chemo and XRT. Patient #IV was treated with Chemo. These four patients received/continued Chemo after the diagnosis of pancreatic metastasis. All four patients had liver masses (Patients # I, III, and IV confirmed to be metastatic breast cancer; Patient #II suspected by imaging). In addition, three patients (Patients #I, III and IV) also had histories of metastatic breast cancer involving bone and brain. The main presenting symptoms associated with pancreatic mass in these four patients were abdominal pain and jaundice. By imaging, solitary pancreatic head masses were found in three patients (Patients #I, II, and IV), ranging from 2.4 to 3.7 cm. One patient (Patient #III) had two pancreatic masses (head, 2.6 cm ‐ FNA; body, 2.9 cm ‐without FNA). EUS‐FNAs targeting the pancreatic head masses on the four patients had sufficient diagnostic material on direct smears, which demonstrated loosely cohesive tumor cells with enlarged, hyperchromatic nuclei, occasional prominent nucleoli, and occasional intracytoplasmic vacuoles. Cell blocks were available for immunohistochemistry (IHC) in all of the cases except one (Patient #II) due to limited cells, which had subsequent histology diagnosis on the specimen from Whipple procedure/pancreaticoduodenectomy. IHC showed the tumor cells were positive for CK7, Gata‐3, GCDFP‐15 (focally positive in Patients # II & IV), and negative for CA19.9. The biomarkers in the metastases were the same as those seen in the primary tumors mentioned above, respectively. The cytomorphologic features and immunostaining pattern were consistent with breast primary (Figure 4). Follow‐up revealed three patients (Patients #I, II, II) expired 5.7 years, 2.8 years, and 1 month, respectively, post‐diagnosis of pancreatic metastasis. Patient #IV without prior breast cancer history/diagnosis has been stable for 6 years (Table 3).

**TABLE 3:** Summary of the four female patients with metastatic breast cancers in pancreas diagnosed by FNA

Pt	Age (Y)	Location of pancreatic mass & size by imaging	Dx & biomarkers of primary tumor/metastasis	Treatment for primary tumor	Other metastatic sites	Interval of primary tumor and pancreatic metastasis	Follow‐up time from Dx of pancreatic metastasis
I	62	Head, 3.5 cm	IDC ER+, PR‐, HER2(3+) /Same	Lumpectomy Chemo XRT	Bone, liver, brain	21 Y	5.7 years (died)
II	37	Head, 2.4 cm	IDC Triple negative /Same	Neoadjuvant T‐mastectomy Whipple	Liver (s/p Whipple)	3.3 Y	2.8 years (died)
III	55	Head, 2.6 cm Body, 2.9 cm	IDC Triple negative /Same	Neoadjuvant Intended‐ Lumpectomy XRT	Bone, liver, brain	1 Y	1 month (died)
IV	64	Head, 3.7 cm	IDC ER‐, PR‐, HER2(3+) /Same	Chemo	Bone, liver, brain	Synchronous*	6 years (stable)

Abbreviations: Ca, carcinoma; Chemo, chemotherapy; Dx, diagnosis; Hx, history; IDC, Infiltrating ductal carcinoma; Neoadjuvant, neoadjuvant chemotherapy; Pt, Patient; T, total; XRT, radiotherapy; Y, year(s).

* Breast mass found clinically on patient presentation.

Further analysis of biomarkers in metastatic breast cancers to the pancreas by literature review (detail references listed in discussion section and Table 4) was performed. We found a total of 51 cases including our four cases of metastatic breast carcinomas to the pancreas, with 22 lobular and 29 ductal carcinomas. Only 30 cases including the current 4 cases had biomarkers reported, and the results of biomarkers were summarized in table 4 (not all articles had all three biomarkers reported). The data showed ten cases of triple negative ductal carcinomas metastatic to the pancreas, followed by three lobular carcinomas with ER+, PR+, HER2, three ductal carcinomas with ER+, PR‐, HER2‐, three lobular carcinomas with ER+, PR+, two lobular carcinomas with ER+, and the rest cases consisting of one case of either ductal carcinoma or lobular carcinoma with the rest patterns of biomarkers listed in Table 4.

**TABLE 4:** Summary of reported biomarkers in total 30 cases of metastatic breast carcinoma to the pancreas including reported cases in the literature (from references 11, 13, 23, 24, 25, 26, 27, 28, 29) and current 4 cases (Ca: carcinoma)

Breast Ca	Biomarkers including ER, PR, and HER2					Biomarkers including ER and PR			Biomarkers including only ER	
	Triple +	Triple ‐	ER+ PR+ HER2‐	ER+ PR‐HER2‐	ER‐ PR‐ HER2+	ER+ PR+	ER+ PR‐	ER‐ PR‐	ER+	ER‐
Lobular	0	0	3	0	0	3	1	1	2	1
Ductal	1	10	1	3	1	1	1	1	0	0

## 4 DISCUSSION

Since its introduction, EUS‐FNA has become the preferred method for diagnosing pancreatic masses as it is accurate and safe.^1^, ^7^ In our institution, we have found a drastic increase in the number of pancreas FNAs performed in 2008 and a peak in 2012, which correlates to our adaptation of EUS‐guided in place of CT‐guided FNAs. In our study of 274 pancreatic FNA cases, the most common diagnosis is ductal adenocarcinoma, comprising 85.7% of cases in the category of positive for malignancy. A similar incidence of ductal adenocarcinoma diagnosed by FNA in solid pancreatic neoplasms has been reported in the literature.^3^, ^5^, ^6^

The pancreas is an uncommon site of metastatic disease. In this study, there are 3.7% of the total pancreatic FNAs and 7.5% of cases in category of positive for malignancy diagnosed as metastases. A study of clinical series has reported less than 5% of metastasis to pancreas.^8^ The finding of metastatic disease to the pancreas can be substantially higher in autopsy series, with frequency in up to 43% of autopsies.^9^ Metastatic neoplasms to the pancreas are often associated with widely disseminated disease, but can also occur rarely as the isolated metastasis.^10^ We have found nine of the ten cases of metastatic tumors to the pancreas with involvement of other organ(s) synchronously, and one case of HCC with only solitary metastasis to the pancreas. The most common sites of metastatic breast cancer are bone, lung, brain, and liver. In our study, there are three of four cases of metastatic breast cancer in pancreas already with metastasis in bone, liver, and brain and one case found to have liver metastasis later.

The most common presenting symptom of patients with metastatic disease in pancreas is jaundice as that observed in majority of our patients. It is not uncommon for patients to be asymptomatic and the pancreatic lesions to be discovered on routine imaging studies.^6^ There are two asymptomatic patients found by routine imaging surveillance to have metastatic clear cell RCC and testicular seminoma, respectively, in our study. The interval from the diagnosis of primary tumor to pancreatic metastatic disease varies from several months to 21 years (Tables 2 and 3). The follow‐up time is also variable from the death after 1 month of the diagnosis of pancreatic metastasis to being stable/alive at the time of this report. The longest time is 13 years/being alive in the patient with metastatic clear cell RCC in this study. The longest time of survival reported in patients with pancreatic metastatic clear cell RCC is 15 years.^6^ Another patient with metastatic infiltrating ductal carcinoma of breast (Patient #IV in Table 3) has been stable/alive for 6 years.

It has been shown that the most common primary tumor metastasizes to pancreas is renal cell carcinoma (RCC) in a large scale study by Hou et al.^6^ Forty‐eight cases of clear cell RCC comprises 44.9% of the 107 cases of the metastases to the pancreas.^6^ Hou et al. have reported 6 cases of breast infiltrating ductal carcinoma and one lobular carcinoma in 107 cases of the pancreatic metastases.^6^ In our study, there is only one case of RCC among the 10 cases of pancreatic secondary tumors. On the other hand, we have found four pancreatic metastases (40%) of breast origin. The incidence of breast metastatic disease to the pancreas is typically low in most studies, though autopsy studies show that metastasis to the pancreas is not uncommon in patients with advanced breast carcinoma.^6^, ^12^, ^13^ Our data of increased number of metastatic breast cancer in pancreas may be due to our patient population. Our institution primarily sees underserved patients in the communities, and underserved women are more likely to be diagnosed with breast cancer at a later stage of disease and have worse outcomes compared to other populations.^14^

The diagnoses of metastases in pancreas FNA can be challenging and majority of the diagnoses are based on clinical history, cytology, and IHC on cell blocks. All of the pancreatic metastatic tumors in our study have cell blocks available for IHC, except one breast metastasis case (Patient #II in Table 3) with insufficient material (IHC performed on the resection specimen due to solitary mass in pancreas at the time of Whipple procedure/pancreaticoduodenectomy; liver metastasis developed later). The four metastatic breast cancers in our study are all infiltrating ductal carcinomas (IDCs) treated with various therapeutic modalities (Table 3) (Detail discussion of treatment is beyond the scope of this study). All four metastatic IDCs are positive for GATA3. The resection specimen from Patient #II (Table 3) shows focally positive GCDFP‐15 by IHC, and cell block from Patient #IV (Table 3) also displays focally positive GCDFP‐15 staining. Mammaglobin immunostain has not been available in house and not performed. GCDFP‐15 and mammaglobin, relatively specific markers by IHC for breast cancer, can be negative in breast metastasis due to their low sensitivity.^15^ A more sensitive marker, GATA3, has been found to be positive in both primary and metastatic breast cancer, although GATA3 is multispecific in different types of cancer cells.^16^, ^17^ A study has revealed that GATA3 expressed in 100% of luminal A and luminal B breast carcinomas, and in 43% of triple‐negative breast cancers.^18^ Recent findings have suggested that immunohistochemical stain for trichorhinophalangeal syndrome type 1 (TRPS1) is a more sensitive and specific marker than GATA3 for breast carcinoma, and TRPS1 is especially useful as a diagnostic tool for triple‐negative breast cancers.^19^

Breast cancer biomarkers, such as ER, PR, and HER2 can also be used in the diagnosis of metastatic breast cancer to the pancreas. However, it is not uncommon to see the conversion of hormone receptors from the primary sites (up to 36%) and the diagnosis cannot be ruled out by negative staining.^20^, ^21^, ^22^ Based on our search of literature in PubMed^13^, ^23^, ^24^, ^25^, ^26^, ^27^, ^28^, ^29^ and the literature review performed by Apodaca‐RuedaI et al. in 2019^11^ on the topic of pancreatic metastases from breast cancer, there are total 51 reported cases including our four cases of metastatic breast carcinomas to the pancreas, with 22 lobular and 29 ductal carcinomas. Among these 51 cases, 30 cases including current 4 cases have reported biomarkers. Table 4 displays the detail information of biomarkers on these 30 cases. The most common pattern of biomarkers is triple negative (ER‐, PR‐, and HER2‐) in ductal carcinoma, comprising 33.3% (10/30 cases) of the cases with reported biomarkers. The data in Table 4 is the first analysis of biomarkers in metastatic breast carcinoma to the pancreas by literature review. It demonstrates that the triple negative ductal carcinoma is the most common metastatic breast carcinoma to the pancreas.

In conclusion, upon retrospectively analyzing FNAs of primary and secondary tumors in the pancreas collected in 10 years in our institution, we have summarized the clinicopathological features including follow‐up of the metastatic cancer to the pancreas. We have found breast carcinoma is the most common secondary pancreatic neoplasm in our patient population. Triple negative breast ductal carcinoma is the most common tumor among the metastasis of breast carcinomas to the pancreas. To the best of our knowledge, this study is the first report with a literature review focusing on biomarkers of metastatic breast cancer to the pancreas.

## AUTHOR CONTRIBUTIONS

**Maria Y Chen:** Data curation (equal); formal analysis (equal); investigation (equal); project administration (equal); resources (equal); validation (equal); visualization (equal); writing – original draft (equal); writing – review and editing (equal). **Neda Zarrin‐Khameh:** Data curation (equal); formal analysis (equal); investigation (equal); resources (equal); validation (equal); visualization (equal); writing – review and editing (equal). **Ya Xu:** Conceptualization (equal); data curation (equal); formal analysis (equal); investigation (equal); methodology (equal); project administration (equal); resources (equal); supervision (equal); validation (equal); visualization (equal); writing – original draft (equal); writing – review and editing (equal).

## FUNDING INFORMATION

This study has no funding support.

## CONFLICT OF INTEREST

The authors have no conflict of interest. The current study was approved by the Ethical Committee/Institutional Review Board (IRB). A waiver/exempt of patients' written informed consents was granted by the Ethics Committee/Institutional Review Board.

## Data Availability

The data that support the findings of this study are available from the corresponding author upon reasonable request.
